# The mediating role of life satisfaction in the relationship between depression, anxiety, stress and burnout among Portuguese nurses during COVID-19 pandemic

**DOI:** 10.1186/s12912-022-00958-3

**Published:** 2022-07-18

**Authors:** Vera Martins, Carla Serrão, Andreia Teixeira, Luísa Castro, Ivone Duarte

**Affiliations:** 1 CINTESIS - Center for Health Technology and Services Research, Faculty of Medicine, University of Porto, Porto, Portugal; 2 School of Education of Polytechnic of Porto, Porto, Portugal; 3 Centre for Research and Innovation in Education (inED), School of Education, Polytechnic of Porto, Porto, Portugal; 4 MEDCIDS - Department of Community Medicine, Information and Decision in Health, Faculty of Medicine, University of Porto, Porto, Portugal; 5 ADiT-LAB, Polytechnic Institut of Viana Do Castelo, Rua Escola Industrial E Comercial Nun’Álvares, 4900-347 Viana do Castelo, Portugal; 6 School of Health of Polytechnic of Porto, Porto, Portugal

**Keywords:** COVID-19, Life satisfaction, Mediating, Burnout, Stress, Anxiety, Depression, Nurses

## Abstract

**Background:**

The COVID-19 pandemic had a large consequence on healthcare systems, increasing the risks of psychological issues in health professionals. Nurses, in particular, have been exposed to multiple psychosocial stressors and struggled with intensive work, insufficiency of resources and uncertainty in the face of an unknown disease. Life satisfaction might protect nurses from the consequences of chronic stress. The aim of this study was to explore the mediating role of satisfaction with life in the relationship between depression, stress, anxiety and burnout (personal, work-related, and client-related).

**Methods:**

A cross-sectional, descriptive, correlational study design was performed, using an online questionnaire distributed via social networks. A total of 379 nurses completed the survey, comprising standardized measures of satisfaction with life, resilience (Resilience Scale), depression, anxiety, stress (Depression Anxiety Stress Scales), and burnout (Copenhagen Burnout Inventory Scale). A hierarchical regression model was estimated for each burnout dimension.

**Results:**

Participants showed high levels of work, personal and client-related burnout, 57.3%, 57%, and 35.1%, respectively. More than 70% of the respondents had a normal level of depressive symptoms, 66.8% presented normal level of anxiety and 33.5% of the respondents reported mild, moderate, severe or extremely severe symptoms of stress. The results revealed that life satisfaction partially mediated the association between stress and personal burnout, depression and work-related burnout, and the association between anxiety and client-related burnout in nurses.

**Conclusions:**

The COVID-19 pandemic brought added difficulties for nurses’ work conditions, whereby it became necessary to develop adaptative measures that reduce stressors in work environment and promote nurses’ life satisfaction.

## Background

Nurses are subject to high stress levels in their professional roles that can lead to numerous mental health disorders. With the emergence of the pandemic caused by the COVID-19 virus, the challenges facing nurses are diverse, given that they have a greater number of patients to attend to, they face a disease about which there are still many doubts and uncertainties, and they are faced with organizational issues in the workplace that lead to feelings of fear and insecurity [1–3].

In a recent study with nurses and physicians from Wuhan, it was verified that female nurses who work directly with COVID-19 patients, were generally more vulnerable to depression, anxiety and stress than male nurses, in these particular working conditions [4]. In a sample of 2,014 frontline nurses from two Wuhan hospitals, more than a half of the individuals presented moderate to high burnout [5]. Weilenmann et al. [6], found that female nurses who had direct interaction with COVID-19 patients reported more symptoms of anxiety, depression, and burnout than colleagues who did not, albeit to a smaller extent. In the same way, Sarboozi et al. [7] found that burnout on frontline nurses was significantly higher than their colleagues working in usual wards. However, in some cases, nurses working in a frontline ward could present lower levels of burnout compared with the usual wards’ nurses [8].

Burnout is a psychological syndrome of physical, emotional, and mental exhaustion, caused by the prolonged exposure to high emotional demands in the workplace [9, 10]. Academics have theorized burnout according to potential sources of psychological fatigue such as personal, client, and work-related dimensions [11]. These requirements are usually caused by a combination of very high expectations from the worker and chronic experiences of stress at work [9]. Research shows that the hospital setting is one of the most stressful working environments experienced by health professionals [12], with nurses especially susceptible to burnout syndrome [13]. Some factors indicated as predictors of burnout are job stress [13], caused by work overload [12], low income, work family conflict [12, 13], working environment, and personal characteristics [13]. However, in some studies, working with patients presented itself as a protective factor against emotional exhaustion and stress [12], which may be related to the duty that nurses feel in serving and caring for patients.

More is known about the risk factors that predict mental health problems than the protective factors and tactics that foster positive development for individuals to cope with atypical stress levels. The pandemic of COVID-19 as an extremely adverse life event became a factor that may contribute for the development of mental health problems [5, 6, 8]. It is of great importance to identify the action strategies to deal with this adverse event, promoting positive social relationships and personal support that can work on the mitigation of mental health problems [14, 15]. The personal characteristics of each one, as subjective well-being, may also mitigate the development of mental health problems [16–19].

The subjective well-being is defined under two distinctive angles “a person’s cognitive and affective evaluations of his or her life” [20]. The affective component is usually divided into pleasant affect and unpleasant affect [21], and the cognitive component is referred to as life satisfaction [22]. Life satisfaction refers to a judgmental process, in which individuals assess the quality of their lives on the basis of their individual selected criteria [21, 23].

Research shows that job burnout has a high correlation with subjective well-being and that a high level of job burnout may cause a decline in subjective well-being [19]. Oates et al. [24] found that demographic and workplace factors did not correlate with subjective well-being measure scores, though features as living alone, being male, and aged between 40–49 years were associated with lower mean scores of subjective well-being [24]. Ghazwin et al. [16] found that age, gender, marital status and satisfaction with interpersonal relationships were not significantly associated with life satisfaction; although, poor satisfaction with financial status and work environment, depression, anxiety and stress were the major determinants of satisfaction with life. In fact, Bartosiewicz and Nagórska [25] in Poland concluded that the place where nurses worked significantly influenced the level of life satisfaction, so nurses working in hospitals had higher levels of satisfaction with life more so than nurses working in primary care or outpatient specialist care units. In the same way, nurses working directly with the patients showed a higher level of satisfaction with life [25]. Qu and Wang [19] found that nurses under 30 years of age scored highest on negative emotion; nurses who are married, have more years of nursing practice and get a higher monthly income, scored higher in positive emotion and life satisfaction. Situations in which nurses are subject to high levels of stress, such as the COVID-19 pandemic, require planning for measures to be implemented that will reduce the damage caused by this. Furthermore, the implementation of strategies to promote nurses’ life satisfaction may be of great relevance for the prevention of mental health disorders.

Research shows that there is a negative correlation between life satisfaction and mental health symptoms of depression, anxiety and stress [26, 27] and that high subjective well-being in nurses is correlated with low levels of depression [16, 17], anxiety [28] and burnout [18, 19].

The COVID-19 pandemic has affected the mental health and well-being of nurses, as it exposes them to extremely stressful work conditions. There are not many studies about the relation of the COVID-19 pandemic and the mediating role of life satisfaction in mental health of nurses, whereby we decided to deepen this topic and study the variables that may be related with this. Based on the previous research it was hypothesized that lesser stress, anxiety, depression, and burnout would be associated with higher subjective well-being, as such, this study aims to analyze the mediating role of life satisfaction in the relationships between anxiety, stress, depression, and burnout among nurses.

## Methods

### Study design

This is a descriptive cross-sectional and correlational study. It was a web-based survey implemented in Google Forms platform, from May 9^th^ and June 8^th^, 2020, applied to nurses working in Portugal. This study received approval from the Ethics Committee of Faculty of Medicine of University of Porto (Ref 184/2020 on May 7^th^, 2020) and was in line with the Declaration of Helsinki. All nurses gave their online informed consent previous to the survey. The questionnaire was made available through nurses’ professional organizations, institutional webpages and social media platforms.

### Measures and covariates

The questionnaire included sociodemographic data (sex, age, marital status, years of professional experience, previous medical history, etc.), the Copenhagen Burnout Inventory, the Resilience Scale, the Depression Anxiety and Stress Scale and the Satisfaction with Life Scale.

Burnout was measured by the validated Portuguese version of the Copenhagen Burnout Inventory (CBI) [9, 11], which is a 19-item tool with three subscales: personal, work-related, and client-related burnout. The personal burnout subscale includes six items such as “How often do you feel worn out?” and assesses the feelings of physical, emotional and mental exhaustion and fatigue. The work-related burnout subscale has seven items, for example, “Do you feel burnout because of your work?” and accounts the symptoms that participants attribute to work. The client-related burnout subscale includes six items such as “Does it drain your energy to work with patients?” and measures the feelings that participants attribute to their work with clients or patients. All items are answered on 5-point Likert scale: 1 (always) to 5 (never). The score for each subscale ranges from 0 to 100 and is the average of item scores within the subscale. Scores that are higher than 50 were considered high-level burnout [9, 11]. The three subscales have high internal consistency, in the original version (α = 0.84) [11] and in the Portuguese version (α = 0.86) [9]. In this study, Cronbach’s alphas were 0.92, 0.90, and 0.88 for personal burnout, work-related burnout, and client-related burnout, respectively.

Psychological resilience was obtained by the Resilience Scale [29]. This scale includes 25 items answered with a seven-point Likert scale ranging from strongly disagree (one) to strongly agree (seven). The total score is determined by the total sum of the 25 items, ranging from 25 (low psychological resilience) to 175 (high psychological resilience). The Portuguese version [30] is characterized by high internal consistency, α = 0.89 [30]. In the current study it was obtained a Cronbach’s alpha of 0.96.

The EADS is the Portuguese version of DASS-21 (Depression Anxiety Stress Scale-21) [31, 32] is a 21-item 4-point Likert questionnaire that contains three subscales designed to measure the emotional states of depression, anxiety, and stress. The subscales include seven items using a scale of zero (did not apply to me at all) to three (applied to me very much or most of the time). For each subscale, a total score is calculated by the sum of the 7 items. On the depression subscale the scores are assigned as follows: normal (0–9), mild (10–13), moderate (14–20), severe (21–27), and extremely severe (28 or more). For the anxiety subscale, scores range from normal (0–7), mild (8–9), moderate (10–14), severe (15–19) to extremely severe (20 or more). In the stress subscale, scores are as follows: normal (0–14), mild (15–18), moderate (19–25), severe (26–33), and extremely severe (34 or more) [31]. In this study, the Cronbach’s alphas were: 0.90, 0.88, and 0.92 for the depression, anxiety, and stress subscales, respectively.

The Portuguese version of the Satisfaction with Life Scale (ESV [Portuguese version]; SWLS [English version]) [33, 34] was validated by Simões [34] and was used to measure participants' life-satisfaction. This is a five-item tool that include items such as “If I could live my life over, I would change almost nothing”, with a five-point Likert scale that measures an individual’s global judgment regarding life satisfaction and ranges from strongly disagree (one) to strongly agree (five) [34]. The ESV has a possible range of 5 (lower satisfaction), to 25 points (higher satisfaction with life). The versions of this scale are characterized by high internal or acceptable consistency, in the original version: α = 0.87 [33] and in the Portuguese version, α = 0.77 [34]. In this study, the Cronbach’s alpha was 0.85.

### Data analysis

Data analysis was performed using Jamovi software (datalab.CC, Sydney, Australia) and SPSS® Statistics (version 27, IBM, Armonk, NY, USA). Categorical variables were presented using absolute and relative frequencies. Quantitative variables were described by the mean and the respective standard deviation (SD) if normally distributed or by the median (Mdn) and the respective interquartile interval (Q_1_; Q_3_) if non-normally distributed. The normality of distributions was verified by observation of the respective Q-Q plots. Correlation between variables was analyzed by Spearman coefficient. Internal consistency of the subscales was verified using Cronbach’s alpha (α). For each outcome of the burnout subscales (personal burnout, work-related burnout, and client-related burnout), a separated multiple linear regression was performed. To decide which independent variables to include in each multiple regression, simple linear regressions were performed with each of the following variables: sex, marital status (married or in a nonmarital partnership; single or divorced or separate or widowed), parental status (no children; ≤ 12 years old; > 12 years old), educational level (graduated; postgraduate), lives with a person at risk for COVID-19 infection (no/yes), years of professional experience (≤ 5 years; 6–15 years; > 15 years), diagnosed health problem (no/yes), direct contact with infected people (no/yes), resilience, depression, anxiety, stress and satisfaction with life scale. All variables that have correlation with the outcomes at *p* ≤ 0.20 in a simple regression were included in the multiple linear regressions. Then the variables were removed in descending order of the p-value, until only the significant variables were maintained in the final multiple models. Stress was the variable that proved to be associated with personal burnout and that met the requirements of mediation, considering life satisfaction as a mediating variable. The same was found between depression and work-related burnout and between anxiety and client related burnout. Hierarchical regression models were estimated to examine the mediating role of satisfaction with life in the relationship between stress and personal burnout; depression and work-related burnout; and anxiety and client-related burnout.

Standardized estimates (β), F statistics, determination coefficient (R^2^), and R^2^-changes (ΔR^2^) for each step were provided. Multicollinearity was verified through tolerances and variance inflation factors ranges, then the Sobel test was pursued to assess the mediation effect. Values of *p* ≤ 0.05 were considered significant.

## Results

### Sample characteristics

We received responses from 409 participants, but 30 of them did not fully complete the questionnaires and were removed from the analysis. The study population comprised 379 nurses in the Portuguese health system.

The sample consists mainly of female nurses 332 (87.6%). Regarding age, nurses had an age range between 22 and 65 years old, and the average age was 41 years old.

The participants worked mainly in primary healthcare (n = 139, 36.7%), 58 in inpatient services with no COVID-19 patients (15.3%), 49 in services with no inpatient wards (12.9%), 44 in emergency services (11.6%), 29 in COVID-19 inpatient areas exclusively (7.7%), 27 in intensive care units (7.1%), 20 in palliative care units (5.3%) and 13 in operating rooms (3.4%). From the participants, 123 (32.5%) had health problems and 37 (9.8%) stated they had started medication for health problems during COVID-19 pandemic (Table 1).

**Table 1:** Characteristics of participants (*n* = 379)

Characteristics	*n*	%
Sex		
Female	332	87.6
Male	47	12.4
Marital status		
Married/nonmarital partnership	240	63.3
Divorced, separated, single, widowed	139	36.7
Parental status		
Yes, with 12 years old or less	129	34
Yes, older than 12 years old	103	27.2
No	147	38.8
Education level		
Graduated	275	72.6
Postgraduate	104	27.4
Professional experience		
Five years or less	48	12.6
From 6 to 15 years	114	30.1
More than 15 years	217	57.3
Direct contact with infected people		
Yes	160	42.2
No	219	57.8
Lives with a person at risk of COVID-19 infection		
Yes	145	38.3
No	234	61.7
Diagnosed health problems		
Yes	123	32.5
No	256	67.5
Anxiety		
Normal	253	66.8
Mild	28	7.4
Moderate	49	12.9
Severe	15	4.0
Extremely severe	34	9.0
Depression		
Normal	277	73.1
Mild	39	10.3
Moderate	39	10.3
Severe	11	2.9
Extremely severe	13	3.4
Stress		
Normal	252	66.5
Mild	43	11.3
Moderate	40	10.6
Severe	31	8.2
Extremely severe	13	3.4
	Mean	SD
Personal burnout	52.7	19.9
Work- related burnout	52.6	19.2
Client-related burnout	37.8	21.4
	Mdn	Q1; Q3
Resilience	137	123;146
Life satisfaction	18	15;19

*Abbreviations*: *SD *standard deviation, *Mdn* median, *Q1* first quartile, *Q3 *third quartile

More than 70% of the respondents had a normal level of depressive symptoms while 2.9% and 3.4% of the respondents presented severe and extremely severe symptoms, respectively. The majority of the respondents (66.8%) presented normal levels of anxiety, followed by moderate symptoms (12.9%) and severe or extremely severe symptoms (13%). From the sample, 33.5% of the respondents reported mild, moderate, severe or extremely severe symptoms of stress.

In general, participants showed high levels of work, personal and client-related burnout, 57.3%, 57%, and 35.1%, respectively. Nurses also showed high satisfaction with life with a median [Q_1_; Q_3_] of 18 [15; 19] points.

Burnout subscales, particularly personal and work-related burnout were significantly associated with anxiety, depression and stress, with Spearman’s rho correlations ranging from 0.51 to 0.60 (*p* < 0.001) as shown in Table 2. Our results showed that satisfaction with life is negatively correlated with all subscales of burnout, meaning that higher levels of life satisfaction are associated with lower levels of burnout.

**Table 2:** Correlations between burnout, anxiety, stress, depression, resilience and life satisfaction measures represented by Spearman’s rho values

	CBI personal	CBI work	CBI client	DASS depression	DASS anxiety	DASS stress	Resilience
CBI work	.781	-	-	-	-	-	-
CBI client	.470	.568	-	-	-	-	-
DASS depression	.521	.600	.383	-	-	-	-
DASS anxiety	.513	.540	.327	.684	-	-	-
DASS stress	.555	.583	.331	.758	.754	-	-
Resilience	-.303	-.295	-.281	-.332	-. 294	-.297	-
SWLS	-.320	-.424	-.356	-.433	-.319	-.318	.315

*Abbreviations*: *CBI* Copenhagen Burnout Inventory, *DASS* Depression Anxiety and Stress Scales, *SWLS* Satisfaction with Lif

All the coefficients are significant at 1% level

### The Mediating Role of Satisfaction with life in the Relationship between Stress and Personal Burnout

In the first step, all the independent variables considered associated with personal burnout (sex, parental status, and anxiety) were adjusted in a multiple linear regression. The obtained model (Table 3) showed a positive association between personal burnout and stress, explaining 5% of personal burnout data variance (β = 0.37, *p* < 0.001), and a negative association between personal burnout and satisfaction with life, accounting for an increase of 2% in the explained variance (β = -0.16, *p* < 0.001).

**Table 3:** Hierarchical linear regression analysis results (outcome variables: personal burnout, work-related burnout, and client-related burnout)

Outcome	Variables	Step 1 (β)	Step 2 (β)	Step 3 (β)
**Personal burnout**	**Sex**			
	*Male*	*Reference*		
	*Female*	0.31*	0.33**	0.32**
	**Parental status**			
	*No*	*Reference*		
	*Yes,* < = *12 years old*	0.26**	0.25**	0.24**
	*Yes,* > *12 years old*	-0.02	0.04	0.00
	**Anxiety**	0.58**	0.28**	0.26**
	**Stress**		0.37**	0.33**
	**Satisfaction with life**			-0.16**
	**F**	53.0**	51.7**	46.9**
	**R**^**2**^	0.36	0.41	0.43
	**ΔR**^**2**^	-	0.05	0.02
**Work-related burnout**	**Anxiety**	0.26**	0.13	0.15*
	**Stress**	0.40**	0.31**	0.30**
	**Depression**		0.25**	0.15*
	**Satisfaction with life**			-0.22**
	**F**	123.1**	89.1**	77.7**
	**R**^**2**^	0.40	0.42	0.45
	**ΔR**^**2**^	-	0.02	0.04
**Client-related burnout**	**Sex**			
	*Male*	*Reference*		
	*Female*	-0.32*	-0.35*	-0.36**
	**Professional experience**			
	*5 years or less*	*Reference*		
	*From 6 to 15 years*	-0.15	0.02	-0.02
	*More than 15 years*	-0.37*	-0.22	-0.31*
	**Resilience**	-0.26**	-0.19**	-0.14**
	**Anxiety**		0.29**	0.20**
	**Satisfaction with life**			-0.27**
	**F**	9.37**	14.77**	18.3**
	**R**^**2**^	0.09	0.17	0.23
	**ΔR**^**2**^	-	0.07	0.06

*Abbreviations*: *β* Standardized estimates, *F*  statistics, *R*^*2*^ determination coefficient, *ΔR*^*2* ^R^2^ changes. *p<0.05; ** *p*<0.001

**Fig. 1:**
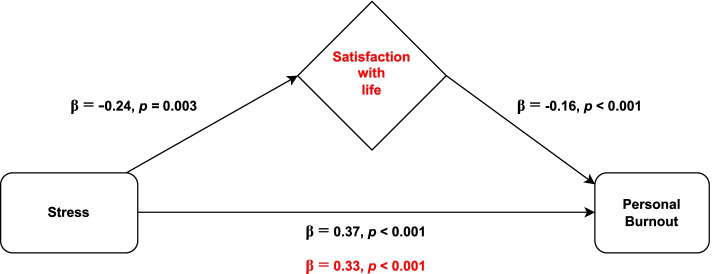
Representative scheme of the mediating role of satisfaction with life in the relationship between stress and personal burnout. Changes in beta weights when the mediator is present are highlighted in red

Given that the absolute value of the stress’s standardized regression coefficient (β) reduced from 0.37 to 0.33 after the inclusion of satisfaction with life in the model (Sobel test, z = 2.33, *p* = 0.020), satisfaction with life was shown to have a partial mediating role in the association between stress and personal burnout (Fig. 1). Multicollinearity was not problematic since tolerance range was 0.586–0.989 and variance inflation factors varied between 1.01 and 1.71. As shown in the figure above, satisfaction with life mediated approximately 11% of the relationship between stress and personal burnout.

### The Mediating Role of Satisfaction with life in the Relationship between Depression and Work-related Burnout

In the first step, all the independent variables considered associated with work-related burnout (anxiety and stress) were adjusted in a multiple linear regression. The obtained model (Table 3) showed a positive association between work-related burnout and depression, explaining 2% of work-related burnout data variance (β = 0.25, *p* < 0.001), and a negative association between work-related burnout and satisfaction with life, accounting for an increase of 4% in the explained variance (β = -0.22, *p* < 0.001).

**Fig. 2:**
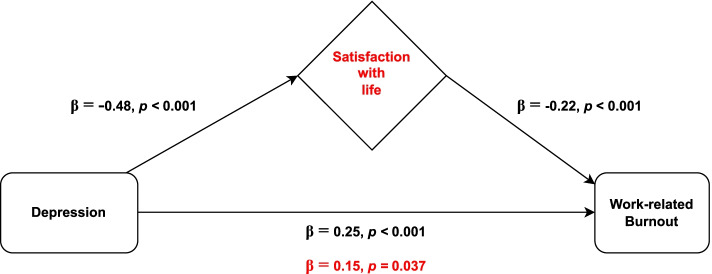
Representative scheme of the mediating role of satisfaction with life in the relationship between depression and work-related burnout. Changes in beta weights when the mediator is present are highlighted in red

Given that the absolute value of the depression’s standardized regression coefficient reduced from 0.25 to 0.15 after the inclusion of satisfaction with life in the model (Sobel test, z = 3.85, *p* < 0.001), satisfaction with life showed to have a partial mediating role in the association between depression and work-related burnout (Fig. 2). Multicollinearity was not problematic since tolerance range was 0.272–0.795 and variance inflation factors varied between 1.26 and 3.68. As shown in the figure above, satisfaction with life mediated approximately 40% of the relationship between depression and work-related burnout.

### The Mediating Role of Satisfaction with life in the Relationship between Anxiety and Client-related Burnout

In the first step, all the independent variables considered associated with client-related burnout (sex, professional experience and resilience) were adjusted in a multiple linear regression. The obtained model (Table 3) showed a positive association between client-related burnout and anxiety, explaining 7% of client-related burnout data variance (β = 0.29, *p* < 0.001), and a negative association between client-related burnout and satisfaction with life, accounting for an increase of 6% in the explained variance (β = -0.27, *p* < 0.001).

**Fig. 3:**
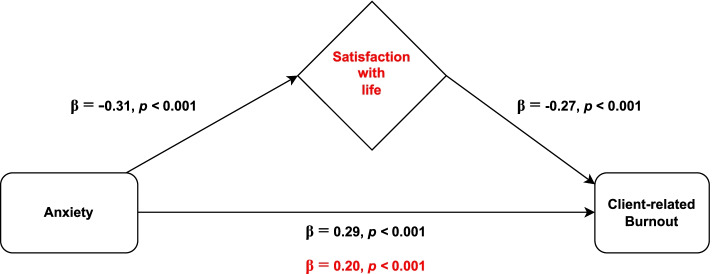
Representative scheme of the mediating role of satisfaction with life in the relationship between anxiety and client-related burnout. Changes in beta weights when the mediator is present are highlighted in red

Given that the absolute value of the anxiety’s standardized regression coefficient reduced from 0.29 to 0.20 after the inclusion of satisfaction with life in the model (Sobel test, z = 4.11, *p* < 0.001), satisfaction with life was shown to have a partial mediating role in the association between anxiety and client-related burnout (Fig. 3). Multicollinearity was not problematic since tolerance range was 0.907–0.994 and variance inflation factors varied between 1.01 and 1.10. As shown in the figure above, life satisfaction mediated approximately 31% of the relationship between anxiety and client-related burnout.

## Discussion

The purpose of this study was to analyze the potential mediating role of life satisfaction in the impact of depression, anxiety and stress on burnout (personal, work-related, and client-related) in Portuguese nurses during COVID-19. A preliminary analysis showed a significant positive correlation between depression, anxiety, stress and burnout dimensions. These data are in line with the literature review and studies that supports that these emotional states are predictors of burnout [35–37]. In addition, we observed significant negative correlations between life satisfaction and mental health symptoms of depression, anxiety and stress, which was consistent with previous studies [16–19, 26–28]. Nurses who reported having higher levels of depression, anxiety, stress and burnout reported lower levels of satisfaction with life.

Of particular interest are the differential relationships found between negative mental states (depression, anxiety and stress) and the three burnout dimensions through life satisfaction. These data support the tripartite model [31], according to which psychological disorders are dimensional and not categorical. Depression, according to this model, is characterized mainly by a loss of self-esteem and motivation and is associated with a perceived low probability of accomplishing life goals that are meaningful to the individual as a person. Anxiety highlights the links between persistent states of anxiety and intense fear responses. Stress, on the other hand, suggests persistent states of arousal and tension, with low levels of resistance to frustration and disappointment [31].

Particularly, nurses who perceived higher level of depression would feel dissatisfied with their life, reported more physical and psychological fatigue and exhaustion related to their work. In fact, work overload, recurrent and prolonged exposure to situations of suffering, the absence of effective means to resolve the situation of illness, the change in paradigm of action due to the demand for physical distance, the substantial increase in deaths, are only some of the factors that may be at the origin of the development of depressive symptoms [38].

With regard to the relationship between depression and burnout, the data from this study reinforces prior findings which states that burnout and depression may develop in tandem [39], or that depression may lead to burnout [40].

On the other hand, nurses perceiving a higher level of stress would feel unsatisfied with life and feel more physical and psychological fatigue and exhaustion as related to their personal life. In fact, stress arises from situations which exceed the physical and/or psychological capacities of individuals [41]. The focus is on the balance between the demands of the situation and the resources (social and personal) that the person has to cope with the demands of that situation. One possible explanation for this is that the unexpected changes in working routines and family dynamics put nurses under strain as a result of the pandemic and can have repercussions on mental health [42]. In fact, our findings suggest that female gender and being a parent of children aged 12 years old or less, are associated with higher levels of personal burnout. Besides which, “safety concerns and fears of getting infected with COVID-19 and putting family members at risk” [43] may be the central causes of stress and burnout.

In turn, nurses who perceive a higher level of anxiety have greater client-related burnout. Anxiety is an aversive emotional response to stress, which results from a threat assessment and is characterized by subjective feelings of worry and apprehension regarding the possibility of physical or psychological harm, often accompanied by increased physiological activation [44]. In this pandemic situation, the fear of being infected, as well as all the uncertainties associated with COVID-19, can justify this data. A study developed by Ahorsu et al. [45] on an Iranian sample showed that the fear of COVID-19 positively correlates with germ aversion, perceived infectiousness and anxiety. Another study found that nurses who presented the largest perceived threat of the pandemic had higher levels of anxiety and social dysfunction [46]. Another possible explanation for this result can be the reduced work experience and low perceived competence to care for patients with COVID-19 that was associated with increased of stress and burnout [43]. In fact, the results of our study reveal that nurses with more than 15 years of professional experience present less risk to develop client-related burnout than those with 5 or less years of experience.

These data showed that males perceived significantly higher client-related burnout in comparison with females. A possible reason for this result can be framed within Eagly's [47] gender role theory, which argues that roles of femininity and masculinity are learned and perpetuated through primary and secondary socialization processes. In this line, it is socially expected that women are more likely to express feelings of emotional and physical fatigue, whereas men should be more likely to switch off and withdraw under stress. Therefore, male nurses tend towards greater depersonalization or distancing oneself psychologically from patients, because they learn to hide their emotions. According to the perspective of Maslach and Jackson [48] “the female role emphasizes caring, nurturance, and concern for other people and their well-being; consequently, women would be less likely to respond to people and their problems in an impersonal and callous manner.”

These results are quite interesting, as they corroborate the idea that burnout is context free and can therefore result from chronic stress from any domain of life, as advocated by several authors [11, 49].

Regarding satisfaction with life acting as a mediator between mental states (depression, anxiety and stress) and burnout (personal, work-related, and client-related), we verified that this mediation is important in explaining how depression, anxiety or stress affects nurse’s well-being. Indeed, life satisfaction seems to be as a substantive variable in psychological health and well-being [50, 51]. Thus, it is important that institutions and nurses’ leaders realize the importance of the implementation and the development of the professionals’ skills and plan specific interventions and psychological support [52]. These interventions could improve the well-being of both nurse leaders and nursing staff [53]. In this way, research shows that some of the measures that could be improved in the workplace are the clinical supervision, the development of work conditions and training [54], the interaction between professionals through personal training [54–56], the development of stress management and working methods [54, 57], the development of a positive attitude in the workplace that allows the professionals to reinterpret negative situations [58].

Also, some interventions focusing more on the professional, may be teaching methods for stress management and resilience-building and behavioral and mental change processes [59, 60] as well as physical exercises like breathing techniques or relaxation skills [54].

### Limitations

This study has some limitations. The study is based on a web‐based survey, disseminated through social networks and email, which might have been influenced by self‐selection bias. The study was carried out during the first wave and is related to only a specific pandemic period, and only enabled us to assess the burnout experienced during a specific period. It would be interesting to replicate this web‐survey in other waves to compare changes in levels of different psychological and occupational variables. Further investigation could employ a longitudinal design which examines the long-term effects of the pandemic in nurses and the level of life satisfaction. No retrospective information was collected. In this sense, we cannot know if depressive symptoms, stress and anxiety, for example, pre-date the pandemic context and whether or not they were exacerbated by the global phenomenon COVID-19. Also, there were a large number of female nurses compared to males, which may underrepresent male nurses. The sample dimension and distribution does not allow us to relate the place where nurses work with the level of life satisfaction as Bartosiewicz et al. [25] found, although it would be interesting to study this relation in future studies.

## Conclusions

Portuguese nurses experienced a high prevalence of physical, emotional, and mental exhaustion. After controlling independent variables (such as sociodemographic and context factors), depression was positively associated with work-related burnout and negatively associated with life satisfaction; anxiety was positively associated with client-related burnout and negatively associated with life satisfaction; stress was positively associated with personal burnout and negatively associated with life satisfaction. Moreover, life satisfaction could partially mediate the relationship between depression, anxiety, stress and different dimensions of burnout. In this sense, when individuals experience several stress and adverse circumstances, such as the COVID-19 pandemic, life satisfaction could protect against distressing events, and can be an operative variable in sustaining mental health. These findings corroborate the role that institutions and nurses’ leaders have in the promotion of better work conditions and strategies that enhance life satisfaction in nurses [61].

## Data Availability

The datasets during or analyzed during the current study are available from the corresponding author on reasonable request.

## Abbreviations

ESV: Escala de satisfação com a vida
SWLS: Satisfaction with life scale
CBI: Copenhagen burnout inventory
EADS: Escalas de ansiedade, depressão e stress
DASS-21: Depression anxiety stress scale-21
α: Cronbach’s alpha
SPSS: Statistical package for the social sciences
IBM: International business machines
NY: New York
USA: United States of America
SD: Standard deviation
Mdn: Median
Q1: First quartile
Q3: Third quartile
P: P-values
Β: Standardized estimates
F: F statistics
R2: Determination coefficient
ΔR2: R2 changes
N: Absolute and relative frequencies
e.g: For example
